# Correlation of Body Parameters and Age with Foot Arch Index and Stabilometric Variables in Physically Active Young Males and Females

**DOI:** 10.3390/sports13090324

**Published:** 2025-09-12

**Authors:** Marco Alessandria, Irene Pivetta, Goran Kuvacic, Nicola Luigi Bragazzi, Sonia Angilletta, Andrea De Giorgio

**Affiliations:** 1 Department of Theoretical and Applied Sciences, eCampus University, 22060 Novedrate, Italy; marco.alessandria@uniecampus.it (M.A.); sonia.angilletta@uniecampus.it (S.A.); andrea.degiorgio@uniecampus.it (A.D.G.); 2 Department of Life Sciences and Systems Biology, University of Turin, 10124 Turin, Italy; 3 Department of Public Health, Experimental and Forensic Medicine, University of Pavia, 27100 Pavia, Italy; irene.pivetta01@universitadipavia.it; 4 Faculty of Kinesiology, University of Split, 21000 Split, Croatia; gorkuv@kifst.hr; 5 Laboratory for Industrial and Applied Mathematics, Department of Mathematics and Statistics, Faculty of Science, York University, Toronto, ON M3J 1P3, Canada

**Keywords:** motor science, balance, proprioceptive control, body sway, stabilometric

## Abstract

Postural stability is influenced by several anthropometric factors as well as age. The aim of this study was to investigate the relationship between body height, body mass, BMI, and arch index with stabilometric parameters in physically active young adults. A total of 169 sport science university students participated in the study. Their arch index and their stabilometric parameters were measured using the P-Walk BTS platform. Spearman’s rank correlations showed a significant positive correlation between body mass and arch index (r = 0.25, *p* = 0.001), and BMI and arch index (r = 0.30, *p* < 0.001). Also, negative correlations were observed between body height and CoP speed (r = −0.22, *p* = 0.004) and CoP distance (r = −0.23, *p* = 0.003), as well as body weight and CoP speed (r = −0.17, *p* = 0.028) and CoP distance (r = −0.19, *p* = 0.015). Age was negatively correlated to postural sway variables (i.e., CoP distance, CoP area, and CoP speed). The findings suggest that, in physically active people, larger anthropometric values may confer stabilizing advantages, potentially due to increased body volume. Also, the significant correlation of age to stability metrics may highlight enhanced proprioceptive integration or adaptive neural mechanisms. The results highlight the importance of individualised proprioceptive training programmes, particularly for athletes of lower body mass or stature. Future research should extend on the results’ potential training and rehabilitative implications.

## 1. Introduction

Postural control is a complex function sustained by neuromuscular and biomechanical systems, and it is particularly relevant during youth, where postural development is still maturing [1]. Among the anatomical structures involved, the foot plays a central role in maintaining both static and dynamic balance [2]. Within this framework, the medial longitudinal arch (MLA) acts as a natural shock absorber, mitigating impact forces and supporting body alignment during movement and stance [3,4,5]. MLA morphology can be quantified using the arch index, which reflects the relative proportion of the metatarsal area [6]. Variations in arch index have been associated with anthropometric parameters such as body mass index (BMI), with higher BMI values generally associated with flattened arches and altered foot loading patterns [7,8,9,10]. Structural adaptations are important as they influence postural control and stability via sensorimotor pathways [11]. For example, excessive flattening of the foot is the result of overuse conditions such as medial tibial stress syndrome and patellar tendon pain [12] or chronic conditions such as low back pain and foot deformities [13,14]. In addition, other anthropometric factors such as weight, height, and age also influence postural sway. Static posturography and centre of pressure (CoP) analysis methods have shown that larger body dimensions affect balance by altering inertial properties and shifting the centre of gravity [15]. For example, Chiari et al. [16] have demonstrated a positive relationship between anthropometrics and sway magnitude, while other authors have found that it appears to be the difference in body composition between the sexes (especially the amount of lean mass in males) that modifies the behavior of the inverted pendulum model [17]. In addition, the literature has emphasised gender-specific neuromuscular strategies, with women generally showing greater instability increases with body height and body mass [18]. However, other authors have not been able to detect any significant difference between the genders [19,20], which warrants further investigation.

There is also evidence that stability increases with age in young adults, possibly due to neurophysiological maturation mechanisms [21,22]. It has been argued that these improvements may be due to improved proprioceptive integration and neural stabilisation of the nervous system following motor experience [23,24]. However, these age-related trends may be different in older people due to neurodegenerative changes [25,26]. Overall, research shows the complex relationship between foot morphology, body mass, and postural control. Nevertheless, based on the findings of previous research, there are still numerous gaps in our knowledge. Given the characteristics of the populations observed in the studies mentioned above, it is unclear whether the effects of anthropometric factors and age would also be evident in a population of physically active young adults. The exact neural mechanisms for improved proprioceptive control in this group are also not well understood. To address these gaps, the present study investigated the relationship between anthropometric measurements and stabilometric and podobarometric measurements in a physically active population with high physical demands, i.e., sports science students. Although previous studies have found relationships between body size, postural stability, and foot shape, the nature and consistency of these relationships are uncertain, particularly in populations with high levels of physical exertion and specific neuromuscular demands. By employing a larger sample size than previous studies [16,18], the present research aimed to shed light on how body dimensions are correlated to both foot morphology and postural stability, also exploring potential gender-associated correlational differences, in physically active young adult male and female sport science students.

## 2. Materials and Methods

### 2.1. Participants

In this cross-sectional study, the data were collected from a dataset consisting of two hundred and three subjects from the bachelor’s degree program in Sciences and Techniques of Motor and Sports Activities whose measurements were carried out at the Sports Science Centre of Turin (Turin, Italy). The evaluation of the level of physical activity of students enrolled in the degree course in Sciences and Techniques of Motor and Sports Activities was carried out by the university department with the questionnaire “International Physical Activity Questionnaire” (IPAQ). It consists of 7 open-ended questions about the activities adults have done in the last 7 days. Weekly PA was measured in minutes per week and minutes per day and then converted to MET minutes (metabolic equivalent × minutes) per week [27,28]. This analysis showed that 80% of the students fell into the “very active” category and the remaining 20% were “normally active” (which identifies a sample with physical activity levels above the average population [29]), despite a pronounced heterogeneity in the type of sporting activity reported (oral communication, unpublished internal report, 4 April 2025). From this dataset, only those individuals who did not have scoliosis, dysmorphic features of the lower limbs, recent fractures of the lower limb skeleton, neurological diseases, recent muscle injuries, or leg length discrepancy at the time of measurement were selected. Therefore, one hundred and sixty-nine of the 203 subjects were included in the analysis. Table 1 presents the demographic characteristics of the recruited sample, revealing a greater proportion of male participants (103 males versus 66 females). The analysis of minimum, maximum, and median values across variables indicated that the age distribution of the overall sample was skewed towards younger individuals, consistent with those typically in the early years of university study (21–23 years). This trend was similarly reflected when stratified by gender. Regarding body mass, the overall distribution mirrored that of age; however, when disaggregated by gender, a right-skewed distribution was observed among female participants compared to their male counterparts. In contrast, the distributions of body height and BMI appeared symmetric in both the overall sample and gender-specific subgroups. Notably, the average BMI across the sample fell within the range typically associated with physically active individuals. We computed the adequacy of the selected sample. We used G*Power analysis 3.1.9.4., by utilizing a linear multiple regression with a fixed model and a single regression coefficient as the statistical test, with the effect size (f^2^) set to 0.25 (calculated based on partial R^2^ obtained from Rosende-Bautista et al. [9]), α set to 0.05, and power set to 0.8. The result of this analysis was a total of 34 participants. Despite this, we employed more participants in order to obtain better model performance. Informed consent was obtained from the adult participants for the data collected in this study. This study was conducted in accordance with the 1964 Declaration of Helsinki and approved by the Institutional Ethics Committee of the University of Split, Split, Croatia (protocol number: 2181-205-02-05-25-0025 approved on 18 February 2025) and all participants gave their informed consent to participate in the study.

### 2.2. Equipment

A stabilometric and baropodometric platform (P-Walk, BTS S.p.A., Garbagnate Milanese, Italy) was used to record the values of balance control and arch index. This platform has a sampling frequency up to 100 Hz. The size of the single module is 675 × 540 × 5 mm, and the height of the sensor surface is 0.7 mm. It has 2304 resistive sensors of 1 cm × 1 cm. The active sampling area is 480 × 480 mm and allows a pressure from 30 to 400 Kpa (300 g/cm^2^). The software that interfaces with the footboard is G-Studio (version 3.3.22.0), which allows the acquisition for the duration of 30/45/60 s in a static position. This device calculates the value of the arch index as the ratio between the area of the middle third of the footprint and the entire footprint area, excluding the toes [6], and the parameters for the assessment of postural stability and foot morphology were validated by Fullin et al., 2022 [30].

### 2.3. Study Design

The tests were carried out as part of the practical lessons of the Bachelor of Sciences and Techniques of Motor and Sports Activities. None of the students had ever performed tests on stabilometric and baropodometric platforms before. The tests were performed in an environment without noise and/or light, which could influence postural control and thus the test on the platform [16,17]. A single test was performed for each participant as this procedure is sufficient to avoid the learning effect in balance tasks for the lower limbs [31]. Once welcomed into the laboratory, each participant was invited to get on the platform barefoot with their feet at a 30° angle [32] and arms relaxed beside their bodies, eyes open and directed towards a target 2 m in front of them at eye level. Each participant performed a single test that lasted 30 s [33,34]. The test began after the operator asked the participant to assume a natural and relaxed upright position for 30 s.

### 2.4. Statistical Analysis

After the distribution of the variables under consideration had been checked using the Shapiro–Wilk normality test (threshold for normality: *p* < 0.05), Spearman’s rank order correlation was carried out. In addition, the presence of outlier values was checked according to the interquartile range method and then, seven values were removed (one in the speed variable, one in the distance variable, and five in the surface variable) in order to avoid unusual influences on the correlation between the variables. The independent variables considered were age (years), body mass (kg), body height (cm), and BMI (kg/m^2^), while the dependent variables determined by the P-walk were CoP speed (mm/s^2^), CoP distance (mm), CoP area (mm^2^), as well as the average arch index calculated from the values of both feet. For this last variable, before calculating the mean value, we verified that there were no significant differences between the arch index values for the right and left foot by performing the Wilcoxon signed-rank test, as the distribution of the arch index values of the right and left foot was asymmetric. The results of this analysis showed no significant differences in the arch index between the right and left foot (z = 0.934; *p* = 0.350). The same procedure was used to examine the gender-specific effects and the analysis was only carried out for the variables that were significantly correlated with each other in the aggregate dataset. The confidence interval of a regression line was depicted because it can provide further information about the distribution of the variables and a range of values into which the mean value of the dependent variable is expected to fall for a given value of the independent variable. The α-level was set to 0.05 and *p*-values < 0.05 were considered significant. Data were analyzed using RStudio (RStudio PBC, RStudio Inc., Boston, United States, version 2024.12.0).

## 3. Results

### 3.1. Body Mass and BMI Correlation with Arch Index and Stabilometric and Baropodometric Variables (CoP Distance, CoP Speed, Arch Index)

**Figure 1:**
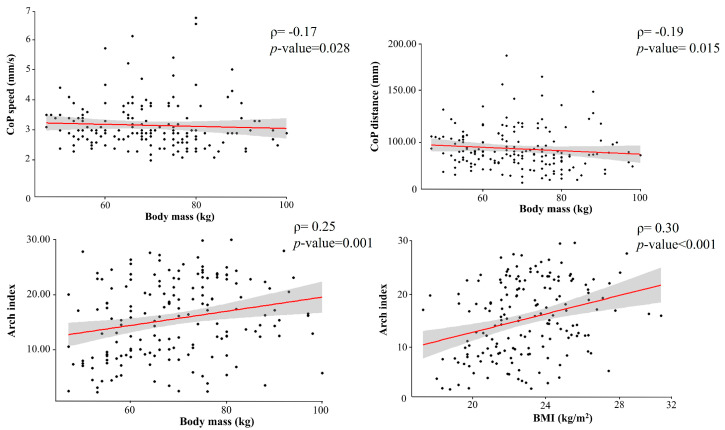
Spearman’s rank correlations between body mass and BMI and the stabilometric and podobarometric variables CoP distance, CoP speed, and arch index. Red line: regression line; Gray area: confidence interval.

Spearman’s rank correlation highlighted a negative correlation between body mass and CoP distance [r(166) = −0.17, *p* = 0.028] and body mass and CoP speed [r(162) = −0.19, *p* = 0.015] while a positive correlation was highlighted between body mass and arch index [r(167) = 0.25, *p* = 0.001] and BMI and arch index [r(167) = 0.30, *p* < 0.001] (Figure 1).

**Figure 2:**
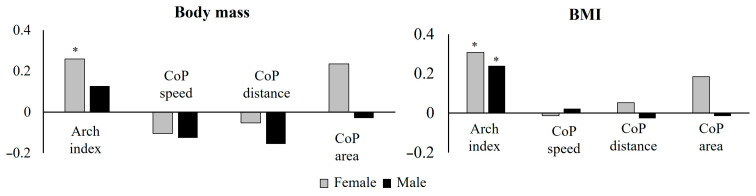
Correlations between body mass and BMI and body and balance variables in female and male groups; * significance level to 0.05.

Between body mass and arch index, Spearman’s rank correlation highlighted a significant positive correlation only in females [r(64) = 0.259, *p* = 0.036], while a significant positive correlation both in females and males was highlighted between BMI and arch index [female: r(64) = 0.307, *p* = 0.012; male: r(101) = −0.240, *p* = 0.014] (Figure 2 and Table S1).

### 3.2. Height Correlation with Stabilometric Variables (CoP Distance and CoP Speed)

**Figure 3:**
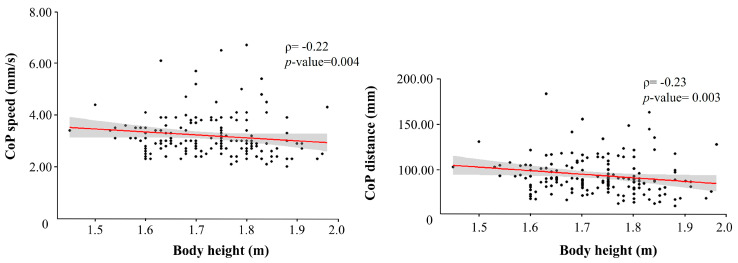
Spearman’s rank correlations between height and the stabilometric variables CoP speed and CoP distance. Red line: regression line; Gray area: confidence interval.

Spearman’s rank correlation highlighted a negative correlation between height and CoP speed [r(162) = −0.23, *p* = 0.003], and height and CoP distance [r(166) = −0.22, *p* = 0.004] (Figure 3).

**Figure 4:**
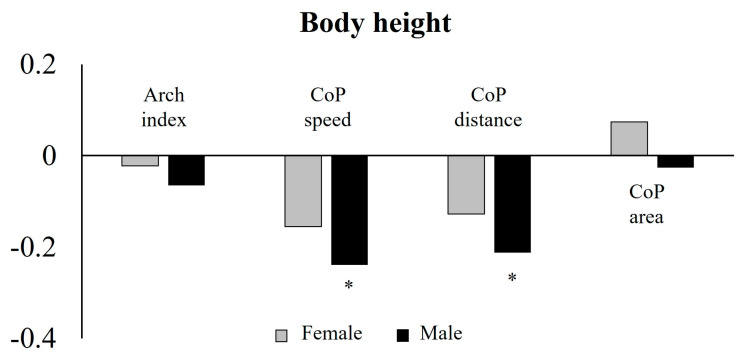
Correlations between height, body and balance variables in female and male groups. * significance level to 0.05.

In the relationship between height, body, and balance variables, Spearman’s rank correlation highlighted a significant negative correlation between height and CoP speed [r(101) = −0.239, *p* = 0.015] and height and CoP distance [r(101) = −0.212, *p* = 0.032] only for males (Figure 4 and Table S1).

### 3.3. Age Effect Correlation with Stabilometric Variables (CoP Distance, CoP Speed, and CoP Area)

**Figure 5:**
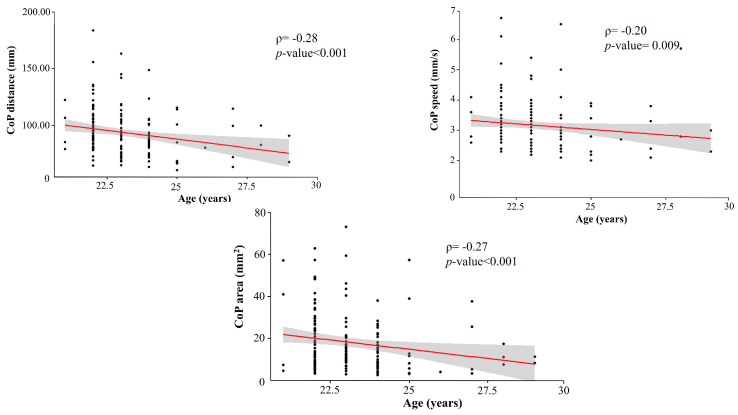
Spearman’s rank correlations between age and the stabilometric variables CoP distance, CoP area, and CoP speed. Red line: regression line; Gray area: confidence interval.

Spearman’s rank correlation showed a negative correlation between age and CoP distance [r(166) = −0.28, *p* < 0.001], age and CoP area [r(166) = −0.20, *p* = 0.009], and age and CoP speed [r(162) = −0.27, *p* < 0.001] (Figure 5).

**Figure 6:**
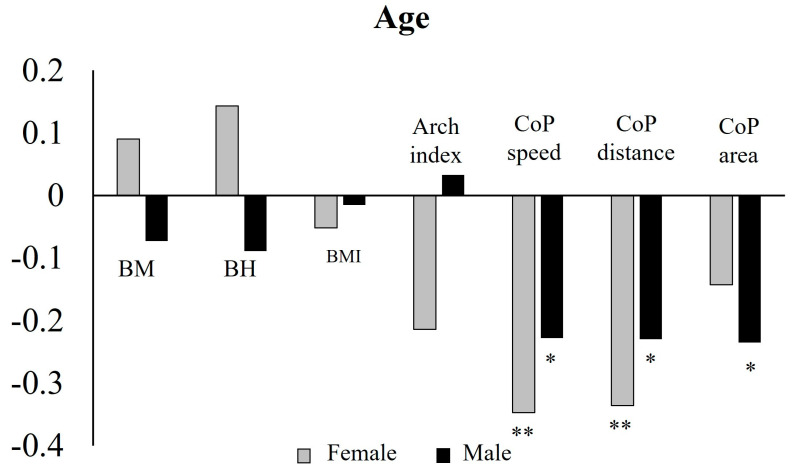
Correlations between age and body and balance variables in female and male groups. BM: body mass; BH: body height; BMI: body mass index; ** significance level to 0.01; * significance level to 0.05.

Spearman’s rank correlation highlighted a significant negative correlation both in females and males between age and CoP speed [females: r(63) = −0.347, *p* = 0.005; males: r(101) = −0.228, *p* = 0.020] and age and CoP distance [females: r(63) = −0.366, *p* = 0.006; males: r(101) = −0.230, *p* = 0.020], while only in males a significant negative correlation was observed between age and CoP area [r(98) = −0.235, *p* = 0.019] (Figure 6 and Table S1).

All remaining non-significant correlations are shown in the Supplementary Materials (Tables S1 and S2).

## 4. Discussion

Our results highlighted that an increase in body size is associated with specific trends in postural control and foot morphology. Specifically, we found a significant positive correlation between BMI and arch index (r = 0.30, *p* < 0.001), confirming trends previously reported in the literature and supporting the hypothesis that physical parameters influence postural function. Specifically, higher body mass, BMI, and height were positively associated with both postural stability and a higher arch index. Moreover, the observed relationship between BMI and arch index is consistent with previous studies [7,8,9,21,22], which have demonstrated a positive association between elevated BMI and increased arch index. However, it is noteworthy that, in line with prior findings [35], no statistically significant correlations emerged between BMI and stabilometric variables within a population demographically comparable to ours. A novel contribution of our study lies in demonstrating that this relationship persists even among individuals with BMI values within the normal range, particularly in a population characterized by a high prevalence of regular physical activity. Since elevated BMI has been linked to foot pathologies such as plantar fasciitis [36], Achilles tendinopathy [37], and hallux valgus [38], we wondered whether the association found in our sample could have a predictive value for osteoarticular pathologies of the foot. van Leeuwen et al. [36] highlighted that these associations appear to be more pronounced in individuals with a BMI exceeding 27 who are also physically inactive. Therefore, to the best of our knowledge, the association observed in our study does not permit us to infer a causal relationship between BMI and foot pathologies in individuals with characteristics similar to our sample, that is, those with an average BMI of 22.9 and who engage in regular physical activity.

However, when analyzing our data in detail, a contrasting trend emerged; indeed, the influence of body size on the stabilometric parameters shows a trend that is in contrast to the results of previous authors. Chiari et al. [16] showed an increase in instability with increasing height and body mass, while Kim et al. [18] confirmed a positive correlation between CoP speed and height only in the female gender, but not between CoP distance and mediolateral CoP speed and body mass, as the correlation was negative.

Our results show a significant negative correlation between body mass and height for the same stabilometric parameters (CoP distance and CoP speed). This finding, which contrasts with the results of the authors cited above, could be related to the significant difference in the sample size of our study compared to that of the authors cited above, which would have allowed us to show a true trend masked by the limited sample size (see Simpson’s paradox). Despite the difference in sample size between our study and previous research, it is important to note that the correlation coefficients obtained have threshold values ranging from low to moderate [39]. This indicates that the strength of the relationships between the variables could be influenced by other aspects. With these aspects in mind, we hypothesize that this outcome is likely attributable to the substantial behavioral variability in postural control, which we believe stems from the wide heterogeneity in the types of sports practiced by participants. Such variability may lead to sport-specific patterns in stabilometric parameters [21]. However, the correlation highlighted in our analysis suggests that the parameters of body mass and height have the same influence on the stabilometric variables that contribute to the increase in postural stability. Therefore, although not measured, we hypothesize that the increase in body volume, given by the joint increase in body mass and height, is more likely to influence stability than a single body factor.

Furthermore, the significant positive correlation between body mass and arch index suggests that the relationship between BMI and arch index is mainly due to the increase in body mass and not to the decrease in height, since the values of Spearman’s rank correlation are similar and no significant correlation was found between height and arch index. In fact, it has been emphasized that excessive weight bearing can lead to a collapse of the arch structure with negative effects on the biomechanics of the foot [40,41].

The unexpected result of this analysis is the significant negative correlation found between age and CoP distance, CoP area, and CoP speed. The interpretation of the result suggests that advancing age within the reference range of our sample helps improve the stability of individuals. Andreeva et al. suggested that postural stability increases until after adolescence and peaks with the full development of all body systems between the ages of 18 and 30 [21]. This phenomenon could be explained by better proprioceptive control, which is probably related to increased body awareness. Leversen et al. [22] hypothesized that part of the positive correlation between motor performance and age may be due to increasing stable connections between groups of neurons. In support of this hypothesis, the authors drew on the ecological theory of neural Darwinism and emphasized that the change in neural connections is based on experience and that certain connections can be strengthened if they are actively used. Furthermore, it is now known that synaptogenesis and its structural changes are dependent on postnatal experience [23,24]. Therefore, we consider it plausible that stability may increase with age. It is important to highlight that, although our findings align with those of Van Humbeeck et al. [26], which indicate that increasing age within the range studied is associated with improved postural stability, the relatively young age of our sample limits the generalizability of these results to older populations. In older individuals, neuromuscular control systems may have already reached peak development and could subsequently decline due to age-related changes in muscle spindle morphology and synaptic transmission, which in turn compromise the reliability of the proprioceptive system [25]. A similar pattern emerged when examining male and female subgroups separately, with a general consistency in trends observed across genders in comparison with the overall sample.

However, there were some interesting differences in body mass, BMI, and height variables that are worth discussing. The positive effect of body mass on the arch index was only significant in females and this result could reflect the different hormonal status between the sexes. The adipose tissue is able to convert the precursors of circulating and stored sex hormones [42]. In particular, increases in adipose tissue and BMI were positively associated with tissue estrogen levels [43]. In addition, estrogens have the effect of increasing tendon and ligament laxity, putting women at greater risk of injury or joint changes [44]. These considerations may explain the positive correlation between body mass and arch index in females but not in males. On the contrary, height was negatively correlated with CoP speed and CoP distance only in males, as well as age and surface. As regards the negative correlation between height and CoP speed and CoP distance, since the tests were performed with eyes open, this result does not suggest a different postural control strategy between the sexes, but rather the different biomechanical properties related to the difference in the height parameter between males and females [16]. Although not significant, the behavior of females also shows a negative correlation between height and CoP speed and CoP distance. Thus, it seems that there is a threshold for the height above which the stabilometric variables are affected, and this should be investigated in further studies. Finally, based on the current state of knowledge, we are unable to offer a plausible explanation for the absence of a correlation between age and support surface area in female participants.

Based on the patterns identified in our sample, the results of this study allow us to reflect on the practical implications of increasing stability with increasing body mass and height and with age.

It is known that in body weightlifting, athletes in the higher body mass classes tend to have endomorphic somatotypic traits that are negatively associated with vertical jump [45,46], whereas mesomorphic and ectomorphic traits have been shown to be associated with other indicators of athletic performance such as explosive power and aerobic capacity [46]. Flexibility, long jump, sprint, and reaction are negatively associated with mesomorphic values, but they are positively associated with ectomorphic values [46]. Based on these considerations, a proprioceptive training program diversified in intensity and volume could be proposed for athletes of different body mass and heights. For athletes who have lower average body mass and height combined with a deficit in strength and balance for the specific demands of the sport practiced, a training program with equipment such as the gym ball could be a valid tool, with exercises performed in a sitting or kneeling position for core control, or with wobble boards or cushions performed in a mono or bipodal position to stimulate performance improvement in overload work.

With regard to the information provided by the present study on the negative correlation between the variables of postural stability and body mass and height, it should be emphasized that this result may represent an initial step toward the development of more tailored training programs that account for both the technical demands of specific sports and the individual characteristics of athletes. For instance, further research in this direction could assist coaches in identifying athletes best suited to particular sports based on their morphometric profiles, as well as in selecting the most effective training methodologies to enhance performance according to individual anthropometric traits. Indeed, low height and body mass values are associated with greater instability, which could be interpreted as a greater predisposition to the development of motor skills such as agility and explosive strength. Conversely, high body mass values are associated with greater postural stability, which could be interpreted as a greater predisposition to develop high maximal strength gradients. Since the results of this study show that subjects have greater stability with increasing body mass and height, we believe that further investigations could identify the modalities of administration of proprioceptive training among individuals with different morphological characteristics.

Ultimately, given the influence of age on stabilometric parameters, we believe it is necessary to consider the practical implications of this result. As mentioned above, it is likely that the strengthening of neural connections caused by previous motor experience is able to implement postural and motor control. In the context of sports performance, this aspect is fundamental as it allows the athlete to improve, modify, or acquire new movement patterns thanks to a solid base of proprioceptive control. While it is well established that each sport induces specific postural adaptations due to the unique movement patterns involved [47,48,49], and pending further research on the relationship between age and postural control in populations with distinct motor skills and training backgrounds, we believe that the findings of this study, highlighting the influence of age on postural stability, support the inclusion of targeted proprioceptive training in athletic development programs. Such interventions may help maintain optimal performance levels throughout an athlete’s developmental trajectory [50]. Although the importance of proprioceptive training is underestimated among coaches, it is known from the literature that this training can improve various aspects of athletic performance: muscle strength, coordination, balance, injury risk reduction, function, and motor skills [50].

Future research should aim to better understand the neural mechanisms underlying the increase in proprioceptive control at the age considered in the present study, which reflects the age group with higher athletic performance. Further studies are needed as well to understand which morphometric indices are best suited to improve performance in different sports disciplines.

## 5. Limitations

Although the study highlighted the relationship between body, stabilometric, and podobarometric variables, we must recognize some limitations. First, given that the recruited population had a BMI within normal limits and a high prevalence of regulating physical activity, it is not possible to definitively conclude that the increase in BMI may lead to plantar arch damage due to the associated increase in arch index. Secondly, given that the level of physical activity in our sample is, on average, higher than the average of the general population, it is possible to generalize our results only to populations whose characteristics overlap with those of the participants we recruited. Finally, due to the young age of our sample, it is not possible to confirm the positive correlation between age and postural stability for older subjects.

## 6. Conclusions

The results of the study confirmed the positive correlation between BMI and the arch index even in a sample of physically active young people, although this relationship cannot be considered a predictor of osteoarticular foot pathologies. Even though this relationship needs to be confirmed by further research, the innovative element of this study is that it finds the same influence of weight and height on stabilometric parameters and hypothesises that greater body volume improves postural stability. The same pattern is also found in postural control with increasing age, suggesting that a strengthening of neural connections caused by previous motor experience is able to implement postural and movement control. Finally, analysis of gender differences revealed a significant relationship between BMI and arch index only in females, suggesting a link between adipose tissue, circulating estrogen levels, and increased ligament and tendon laxity. Conversely, the height variable was negatively correlated with CoP speed and CoP distance only in males, suggesting a threshold for height above which the stabilometric variables are affected. These considerations lead us to reflect on both the importance of a personalised approach to training and the possibility of helping coaches identify the most suitable athletes for specific sports based on their morphometric profiles.

## Figures and Tables

**Table 1:** Demographic characteristics of the sample. N: sample size; SD: standard deviation.

Groups	N	Age	Height (cm)	Body Mass (kg)	BMI
		Mean ± SD	Mean ± SD	Mean ± SD	Mean ± SD
		Min, Max, Median	Min, Max, Median	Min, Max, Median	Min, Max, Median
Overall	169	23.1 ± 1.4	173 ± 9.7	69.0 ± 12.2	22.9 ± 2.4
		21, 29, 23	145, 196, 175	47, 100, 68	17.3, 30.2, 22.7
Female	66	22.9 ± 1.07	164 ± 7.0	59.2 ± 8.0	21.8 ± 2.2
		21, 27, 23	145, 182, 164	47, 82, 58	17.3, 28.0, 21.9
Male	103	23.3 ± 1.7	178 ± 6.9	75.3 ± 10.1	23.6 ± 2.3
		21, 29, 23	164, 196, 178	54, 100, 75	18.0, 30.3, 23.5

## Data Availability

The raw data supporting the conclusions of this article will be made available by the authors, without undue reservation.

## Supplementary Materials

The following supporting information can be downloaded at: https://www.mdpi.com/article/10.3390/sports13090324/s1, Table S1: Correlation matrix in the female and male group. ** significance level to 0.01; * significance level to 0.05; Table S2: *p*-value matrix of the non-significant *p*-value in the total sample. * Significant *p*-value and reference figure; AI: arch index; BMI: body mass index; LSF: length surface function.

## Abbreviations

The following abbreviations are used in this manuscript:

BMI	Body Mass Index
CoP	Center of Pressure
