# Supplementary material for: Correlation of Body Parameters and Age with Foot Arch Index and Stabilometric Variables in Physically Active Young Males and Females
Source: Sports (Basel). 2025 Sep 12;13(9):324. doi: 10.3390/sports13090324 (PMC12474270; doi:10.3390/sports13090324)
Supplement: Supplementary file 1 [file sports-13-00324-s001.zip › sports-3743960-supplementary.pdf]

Correlation matrix in the female group

		Age	Weight	Height	BMI	Arch index	Speed	Distance	Surface
**Age**	Correlation coefficient	1.000	0.090	0.143	-0.052	-0.214	-0.347**	-0.336**	-0.143
	p-value		0.473	0.252	0.676	0.085	0.005	0.006	0.259
**Weight**	Correlation coefficient		1.000	0.571**	0.738**	0.259*	-0.105	-0.053	0.235
	p-value			<0.001	<0.001	0.036	0.406	0.674	0.062
**Height**	Correlation coefficient			1.000	-0.046	-0.022	-0.155	-0.128	0.074
	p-value				0.712	0.861	0.217	0.310	0.559
**BMI**	Correlation coefficient				1.000	0.307*	-0.014	0.052	0.184
	p-value					0.012	0.915	0.683	0.146
**Arch index**	Correlation coefficient					1.000	0.025	0.008	0.045
	p-value						0.846	0.950	0.724
**Speed**	Correlation coefficient						1.000	0.954**	0.641**
	p-value							<0.001	<0.001
**Distance**	Correlation coefficient							1.000	0.682**
	p-value								<0.001
**Surface**	Correlation coefficient								1.000
	p-value								

Table S1 - Correlation matrix in the female group. ** significance level to 0.01; *significance level to 0.05

Correlation matrix in the male group

		Age	Weight	Height	BMI	Arch index	Speed	Distance	Surface
**Age**	Correlation coefficient	1.000	-0.073	-0.089	-0.015	0.033	-0.228*	-0.230*	-0.235*
	p-value		0.463	0.372	0.880	0.744	0.020	0.020	0.019
**Weight**	Correlation coefficient		1.000	0.716**	0.792**	0.127	-0.126	-0.156	-0.030
	p-value			<0.001	<0.001	0.200	0.207	0.115	0.764
**Height**	Correlation coefficient			1.000	0.184	-0.065	-0.239*	-0.212*	-0.026
	p-value				0.063	0.517	0.015	0.032	0.795
**BMI**	Correlation coefficient				1.000	0.240*	0.022	-0.027	-0.015
	p-value					0.014	0.824	0.784	0.878
**Arch index**	Correlation coefficient					1.000	0.082	0.048	-0.003
	p-value						0.408	0.632	0.975
**Speed**	Correlation coefficient						1.000	0.910**	.664**
	p-value							<0.001	<0.001
**Distance**	Correlation coefficient							1.000	0.687**
	p-value								<0.001
**Surface**	Correlation coefficient								1.000
	p-value								

Table S2 - Correlation matrix in the male group. ** significance level to 0.01; *significance level to 0.05
